# Activation of endogenous p53 by combined p19Arf gene transfer and nutlin-3 drug treatment modalities in the murine cell lines B16 and C6

**DOI:** 10.1186/1471-2407-10-316

**Published:** 2010-06-22

**Authors:** Christian A Merkel, Rafael B da Silva Soares, Anna Carolina V de Carvalho, Daniela B Zanatta, Marcio C Bajgelman, Paula Fratini, Eugenia Costanzi-Strauss, Bryan E Strauss

**Affiliations:** 1 Setor de Vetores Virais, Laboratório de Genética e Cardiologia Molecular/LIM 13, InCor, FM-USP, São Paulo, Brasil; 2 Programa de Biotecnologia, ICB-USP, São Paulo, Brasil; 3 Instituto do Milênio-Rede de Terapia Gênica, MCT, São Paulo, Brasil; 4 Departamento de Biologia Celular e do Desenvolvimento, Instituto de Ciências Biomédicas, USP, São Paulo, Brasil

## Abstract

**Background:**

Reactivation of p53 by either gene transfer or pharmacologic approaches may compensate for loss of p19Arf or excess mdm2 expression, common events in melanoma and glioma. In our previous work, we constructed the pCLPG retroviral vector where transgene expression is controlled by p53 through a p53-responsive promoter. The use of this vector to introduce p19Arf into tumor cells that harbor p53wt should yield viral expression of p19Arf which, in turn, would activate the endogenous p53 and result in enhanced vector expression and tumor suppression. Since nutlin-3 can activate p53 by blocking its interaction with mdm2, we explored the possibility that the combination of p19Arf gene transfer and nutlin-3 drug treatment may provide an additive benefit in stimulating p53 function.

**Methods:**

B16 (mouse melanoma) and C6 (rat glioma) cell lines, which harbor p53wt, were transduced with pCLPGp19 and these were additionally treated with nutlin-3 or the DNA damaging agent, doxorubicin. Viral expression was confirmed by Western, Northern and immunofluorescence assays. p53 function was assessed by reporter gene activity provided by a p53-responsive construct. Alterations in proliferation and viability were measured by colony formation, growth curve, cell cycle and MTT assays. In an animal model, B16 cells were treated with the pCLPGp19 virus and/or drugs before subcutaneous injection in C57BL/6 mice, observation of tumor progression and histopathologic analyses.

**Results:**

Here we show that the functional activation of endogenous p53wt in B16 was particularly challenging, but accomplished when combined gene transfer and drug treatments were applied, resulting in increased transactivation by p53, marked cell cycle alteration and reduced viability in culture. In an animal model, B16 cells treated with both p19Arf and nutlin-3 yielded increased necrosis and decreased BrdU marking. In comparison, C6 cells were quite susceptible to either treatment, yet p53 was further activated by the combination of p19Arf and nutlin-3.

**Conclusions:**

To the best of our knowledge, this is the first study to apply both p19Arf and nutlin-3 for the stimulation of p53 activity. These results support the notion that a p53 responsive vector may prove to be an interesting gene transfer tool, especially when combined with p53-activating agents, for the treatment of tumors that retain wild-type p53.

## Background

For those tumors that retain wild type p53 (p53 wt), maintenance of the tumor phenotype depends on its ability to hold p53 wt in an inactive form even long after the initial transformation events [1-4]. These studies showed that reactivation of p53 impeded tumor growth, rekindling interest in this treatment approach. However, the induction of p53 function may meet with significant barriers, such as loss of p19Arf (p14ARF in humans) or over-expression of mdm2 (HDM2 in humans).

For melanomas, p53 wt is present in 90% of cases, over-expression of HDM2 is found in 56% cases [5,6] and loss of the CDKN2A locus (where p14ARF resides) occurs in some 50% of primary melanomas [7]. In comparison, primary human gliomas retain p53 wt in 70% of cases [8], the loss of p14ARF appears to be a reciprocal event [9] and 50% of cases over-express HDM2 [10]. These findings indicate that maintenance of inactivated p53 wt in melanomas and gliomas is directly associated with p14ARF/HDM2 status and that this axis may serve as a therapeutic target [11].

Re-activation of p53 may be achieved by gene transfer or pharmacologic approaches. Gene transfer studies have shown that introduction of p14ARF can activate p53 [12-14]. Drug treatment with nutlin-3, a small molecule compound that specifically blocks the interaction of mdm2/HDM2 with p53, results in the protection of p53 from proteolytic degradation [15,16]. However, not all tumor cells with p53wt are sensitive to nutlin-3 treatment, an effect thought to be related to mdmx/HDMX activity [17,18]. To the best of our knowledge, there are no reports in the literature where p19Arf gene transfer was combined with nutlin-3 drug treatment. Since p19Arf has been shown to interact with and inhibit mdmx [19,20], this may provide an additional mechanism for establishing nutlin-3 sensitivity.

We present here the use of a p53-responsive retroviral vector, pCLPG, for the transfer of the p19Arf cDNA. We have shown previously that pCLPG provided p53-specific expression that was, in some cells, stronger than the expression level of the parental, non-modified vector, pCL [21]. When the pCLPG vector was employed for transfer of the p53 cDNA, a positive feedback regulatory mechanism was established that both drove vector expression and also blocked tumor cell proliferation [22]. Here the pCLPG vector was used to introduce the p19Arf cDNA in cells harboring endogenous p53 wt in order to explore the interplay between the vector, the transgene and cellular p53.

The pCLPGp19 virus was used to treat cell lines that carry wild type p53, B16 (mouse melanoma, p19Arf-null) and C6 (rat glioma, p19Arf-null). Since p19Arf can work in conjunction with the p53 pathway, we proposed that introduction of exogenous p19Arf may functionally activate endogenous p53, impacting both vector expression and tumor cell proliferation. These gene transfer experiments were performed with or without additional drug treatment (doxorubicin, nutlin-3) to determine if combined genetic and pharmacologic therapies could overcome the barriers to p53 function in these cells.

## Methods

### Construction of vectors

The construction of the pCLPG vector containing eGFP has been described previously [21,22]. In these studies, we showed that expression from the pCLPG-ΔU3 construct offered the strongest response to p53 and was chosen for further study, though it is referred to here simply as pCLPG. The p19Arf cDNA (kindly provided by Charles Sherr, St. Jude's Children's Hospital, Memphis, TN) was first subcloned into pBluescript (Stratagene) and re-isolated as an 800-bp *Bam*HI fragment. This fragment was then inserted in the *Bam*HI site of the pCLPG vector.

### Cell culture and lines

The adenovirus-transformed, human embryo kidney cell line 293T [23] and the rat glioma cell line C6 (ATCC CCL-107) were maintained in DMEM (Invitrogen, USA) supplemented with 10% bovine calf serum (BCS, HyClone, Logan, UT, USA), 100 μg/ml gentamycin, 50 μg/ml ampicillin, and 2.5 μg/ml fungizone, at 37 °C, in a humidified atmosphere of 5% CO_2_. The mouse melanoma cell line B16F10 (B16, ATCC CRL-6475) was cultured as above, except using RPMI (Invitrogen, USA).

### Virus production

To produce virus-containing supernatant, the appropriate viral vectors were co-transfected in 293T cells as described [24], except using pCMV-gag-pol and pCMV-VSVg packaging vectors (kindly provided by Richard Mulligan, Harvard Medical School, Boston, MA, USA and Jane Burns, University of California, San Diego, USA, respectively). The virus-containing supernatant was collected 24 hours post-transfection, centrifuged for 5 minutes, 1000 × g, and the supernatant was aliquoted and stored at -70 °C. Titration was performed either by end-point dilution determined by G418 resistance or, when possible, by counting eGFP-positive cells by flow cytometry. These protocols have been described previously [25]. Typical titers were in the range of 1 to 5 × 10^6 ^colony forming units (cfu)/ml.

### Growth curve

The indicated cell type was plated, 7.5 × 10^5 ^cells/6 cm dish, and transduction was initiated the following day with equal quantities and concentrations of the indicated viruses (1 × 10^6 ^particles in 1.5 ml) or mock transduced in the presence of polybrene, 8 μg/ml. The transduction was allowed to proceed for 8 hours before a second round of transduction was initiated and allowed to proceed overnight. A third round of transduction was initiated the following morning and allowed to proceed for 6 hours. At the end of the transduction, the cells were trypsinized, counted and replated, 1 × 10^4 ^cells of each transduction were plated in each of the wells in a 12-well dish with complete medium (DMEM for C6 or RPMI for B16). Two wells per day, where day 1 represents 24 hours post replating, were trypsinized and counted manually.

### Colony formation assay

The indicated cell type was plated, 2 × 10^5 ^cells/well in 6-well dishes, and transduced the following day with equal quantities and concentrations of the indicated viruses (4 × 10^5 ^particles in 600 μl) or mock transduced in the presence of polybrene, 8 μg/ml. After 4 hours incubation at 37˚C, the virus supernatant was replaced with fresh medium. The next day, cells were harvested, counted, and re-plated at 7.5 × 10^3^, 1.0 × 10^4^, 2.5 × 10^4 ^and 5.0 × 10^4 ^cells/dish in 6 cm dishes. The following day, the medium was replaced with fresh, complete medium (DMEM for C6 or RPMI for B16) containing 800-1000 μg/ml of G418 and cells were incubated until mock transduced cells had died, about 7 days. Then, medium was replaced and colonies allowed to form until clearly visible, usually an additional 7-14 days. Cells were fixed with 0.5% paraformaldehyde and stained with crystal violet. For quantification, the crystal violet was recovered in 10% acetic acid and the absorbance read at 590 nm using a spectrophotometer. The positive control, the colonies resulting from the empty pCLPG vector, was considered as 100%.

### Immunofluorescence detection of p53 and p19Arf

Cells were transduced as per the growth curve assay and then replated on 13 mm round glass coverslips, 5 × 10^4 ^cells/well, in 24 well dishes. Following the final round of transduction, either fresh medium, medium containing 100 ng/ml doxorubicin or medium containing 10 μM nutlin-3 was used and the cells incubated for an additional 24 hours. The cells were fixed with cold methanol, blocked with bovine serum albumin, then probed with a polyclonal antibody for p19Arf (AB-1, Cal-Biochem) followed by an Alexa-488 labeled anti-rabbit secondary antibody. Staining for p53 was performed with a pan-p53 monoclonal antibody (clone G59-12, BD Biosciences) followed by Cy3 labeled anti-mouse secondary antibody. Nuclear staining was performed with Hoechst 33258, 20 μg/ml. Cells were visualized by confocal microscopy at either 20 × amplification (B16) or 20 × plus 4 × zoom (C6).

### Northern blot

For the Northern blots, 2 6-cm dishes of each cell line were transduced as per the growth curve assay. The transduced cells were then treated overnight at 37˚C with complete medium or medium plus 100 ng/ml of doxorubicin. Total RNA was purified using Trizol reagent (Invitrogen Life Technologies, USA) according to the manufacturer's instructions and samples were analyzed as described previously [21].

### Western blot detection of p19Arf

Cells were transduced and treated with drugs as described for the p53 activation assays. Protein lysates were made 24 hours after initiation of drug treatment and western blot analysis was performed. Briefly, RIPA buffer (1% NP-40, 0.1% SDS, 0.5% Sodium Deoxycholate in 1 × PBS) supplemented with complete mini protease inhibitor cocktail (Roche) was used to lyse cells, the protein concentration was determined and then 20 μg was subjected to SDS-PAGE before transfer to Hybond ECL membrane (GE Lifesciences) and probing with an anti-p19Arf antibody (Ab-1, CalBiochem), anti-p21 (sc-756, Santa Cruz Biotechnology) or β-Actin (A5441, Sigma). Secondary antibodies labeled with horse-radish peroxidase were applied and detected with ECL-Plus reagent according to the manufacturer's protocol (GE Lifesciences).

### p53 activation measured in a reporter assay

For the reporter assays, cells were transduced with pCLPG, pCLeGFP or pCLPGeGFP viruses and selected for G418 resistance, as reported previously [26]. Cells were replated, 1 × 10^6 ^cells/6 cm dish, and transduced (as described for the growth curve assays) with the indicated virus. At the end of the transduction, cells were replated at approximately 50% density in 6-well dishes. The medium in duplicate wells was changed the next day (DMEM for C6 or RPMI for B16) or replaced with medium containing 100 ng/ml doxorubicin or 10 μM nutlin-3 (N6287, Sigma, USA). The cells were incubated for 24 hours before harvesting for flow cytometric assessment of eGFP expression and, in parallel, analysis of cell cycle as revealed by propidium iodide staining as described previously[26]. The median intensity of eGFP expression was determined by the FACS software and then normalized considering the positive control (pCLeGFP) as one.

### Cell viability assay

Cells were transduced as described for the growth curve. For the MTT assay, 96-well dishes were seeded with 1 × 10^4 ^cells from each transduction using complete medium (DMEM for C6 or RPMI for B16) in quadruplicate wells. The next day, fresh medium or medium containing the indicated quantities of drug was applied to the dishes. Cells were incubated for an additional 48 hours before determination of cell viability. Plates were incubated with 25 μl of MTT solution (5 mg/ml in 1 × PBS), 37°C during 4 hours. The dish was then removed and the precipitate solubilized by the addition of 100 μl lysis buffer (20% SDS in 50% DMF/2% acetic acid, pH adjusted to 4.7) before analysis using a plate reader at 590 nm.

### Animal model

Procedures and conditions for these experiments were approved by the Scientific and Ethics Committee of the Intituto do Coração (2833/06/128) and Hospital das Clinicas (735/06), University of São Paulo School of Medicine. B16 cells were transduced *ex vivo*, as described for the growth curve assay, with pCLPG or pCLPGp19 virus, in 10 cm dishes. Upon completion of the final round of transduction, the cells were replated in triplicate 10 cm dishes and allowed to reach 80% density before medium was replaced with fresh RPMI containing no drug or RPMI containing 25 ng/ml doxorubicin or 10 μM nutlin-3. The next day, cells were harvested, counted and injected subcutaneously in the flank, 1 × 10^6 ^cells per C57BL/6 mouse (n = 4), in 100 μl PBS. Tumors were allowed to develop during 15 days, then all animals were injected i.p. with 100 mg/kg bromodeoxyuridine, BrdU, maintained for an additional 4 hours, then sacrificed and tumors were collected and analyzed. One half of each tumor was submitted to frozen sectioning while the remaining half was fixed in paraformaldehyde 4% followed by inclusion in paraffin. Histologic sections, 3-5 μm, were prepared and stained with hematoxylin and eosin (HE). Necrotic area was identified visually in 3 sections from each tumor and quantified using ImageJ software (15-20 fields for each section) and the ratio of necrotic/non-necrotic tissue was determined.

BrdU staining was performed using paraffin embedded sections and following the protocol supplied with the BrdU peroxidase staining kit (Zymed laboratories). TUNEL staining was performed using frozen sections and following the protocol supplied with the *In situ *cell death detection kit, Fluorescein (Roche Applied Biosciences).

## Results

### Reliable expression of p19Arf when delivered by the pCLPG retrovirus to cell lines harboring wild type p53

**Figure 1:**
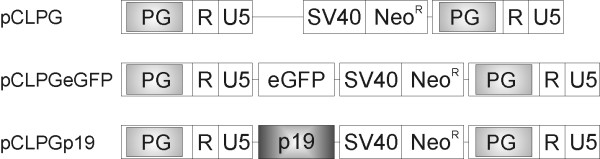
**Schematic representation of the p53-responsive pCLPG retroviral vectors**. These vectors contain a p53-responsive element, called PG, inserted in the retroviral long terminal repeat. This modification results in p53-dependent transgene expression [21,22]. R U5, native regulatory elements of the Moloney Murine Leukemia Virus long terminal repeat; SV40, simian virus 40 promoter; Neo^R^, neomycin phosphotransferase cDNA which confers resistance to the antibiotic G418; eGFP, enhanced green fluorescent protein; p19, mouse p19Arf cDNA.

**Figure 2:**
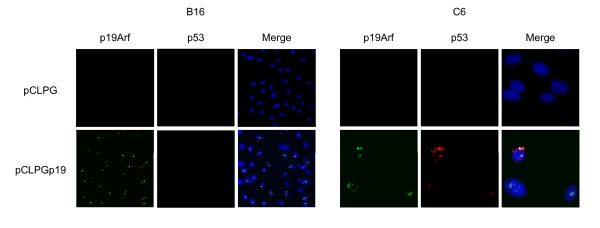
**Exogenous p19Arf detected by immunofluorescence**. Cells were transduced and, 24 hours later, fixed with methanol and exposed to a polyclonal antibody for p19Arf and a monoclonal antibody for p53 and detection with Alexa-488 anti-rabbit and Cy3 anti-mouse secondary antibodies. Cells were then stained with Hoechst 33258. For B16, we show only the 20 × magnification confocal photomicrographs, no staining of p53 was visible at higher magnification. For C6, the appearance of p19Arf at 20 × magnification was comparable to that shown for B16, however endogenous p53 staining was observed in C6. We show C6 at 20 × magnification plus 4 × zoom in order to emphasize p53 staining. All photos for either B16 or C6 were captured using identical settings. Merge refers to the combined images of the green, red and blue channels.

We sought to target endogenous p53 wt to both drive vector expression and inhibit tumor proliferation by including the p19Arf cDNA in the pCLPG retrovirus, a vector that contains a p53-responsive promoter used to control expression of the transgene (**Figure **1). To confirm transgene expression, we first performed immunofluorescence staining of B16 (mouse melanoma, wild-type p53, p19Arf null) and C6 (rat glioma, wild-type p53, p19Arf null) cells transduced with pCLPGp19 or, as a control, with the empty pCLPG retrovirus. For both cell lines, we readily detected exogenous p19Arf localized in the nucleolus only in the presence of the pCLPGp19 virus (**Figure **2). The presence of endogenous p53 was also examined by immunofluorescence staining. The treatment of C6 cells with pCLPGp19 facilitated the detection of endogenous p53 which was localized in close proximity to p19Arf. Similar treatment of B16 did not reveal endogenous p53 (**Figure **2).

**Figure 3:**
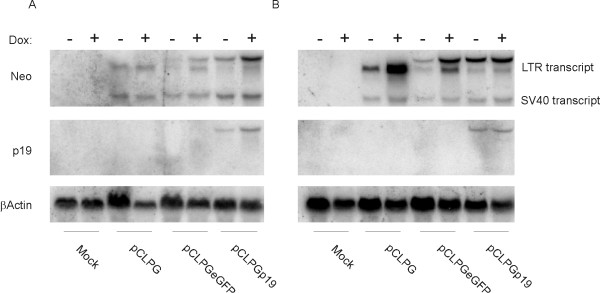
**Northern blot analysis reveals that presence of p19Arf aids pCLPG vector expression**. B16 (A) or C6 cells (B) were mock transduced or transduced with equal quantities of pCLPG, pCLPGeGFP or pCLPGp19 in duplicate dishes. One dish from each duplicate was treated with 100 ng/ml of doxorubicin (Dox) and the other was maintained in fresh medium for 24 hours before harvesting total RNA used to perform the Northern blot analyses. Membranes were probed sequentially using the radiolabeled cDNAs of neomycin phosphotransferase (Neo), p19Arf (p19) or, as a control for loading, βActin.

Northern blots showed that viral expression from the pCLPG vectors in B16 cells was generally weaker than that seen for C6 cells, but the presence p19Arf aided viral expression and viral transcripts were readily detected (**Figure **3). Treatment of the cells with doxorubicin (an inhibitor of topoisomerase II and DNA damaging agent well known for its ability to activate p53) did result in increased viral expression in both cell lines. At least in this assay, the p19Arf transgene appears to have enhanced vector expression.

**Figure 4:**
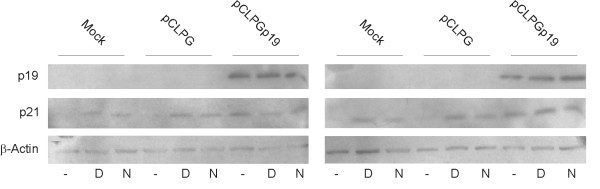
**Western blot analysis of p19Arf and p21 (Cdkn1a) expression upon gene transfer and drug treatments**. B16 (left) and C6 (right) cells were mock transduced or transduced with pCLPG or pCLPGp19 followed by treatment with 100 ng/doxorubicin or 10 μM nutlin-3 (lanes labeled D or N, respectively) or no drug (-). Exogenous p19Arf, endogenous p21 and β-Actin were revealed using specific primary antibodies followed by secondary HRP-conjugated antibody/ECL detection.

Prior to Western blot analysis, B16 and C6 cells were subjected to gene transfer with or without exposure to doxorubicin or nutlin-3 (an inhibitor of mdm2). As seen in **Figure **4, transduction of either cell line with pCLPGp19 yielded readily detectable levels of p19Arf, but the protein level was not increased upon drug treatment. Induction of p21 (Cdkn1a) expression was accomplished by either p19Arf gene transfer or drug treatment. Though p21 is a known p53 target, we cannot rule out its activation by p53-independent mechanisms.

In all, these assays indicate that the expression of p19Arf from the pCLPG vector was reliable in p53 wt-positive cells.

### Proliferation of B16 cells is not altered by treatment with pCLPGp19, yet C6 cells are inhibited

**Figure 5:**
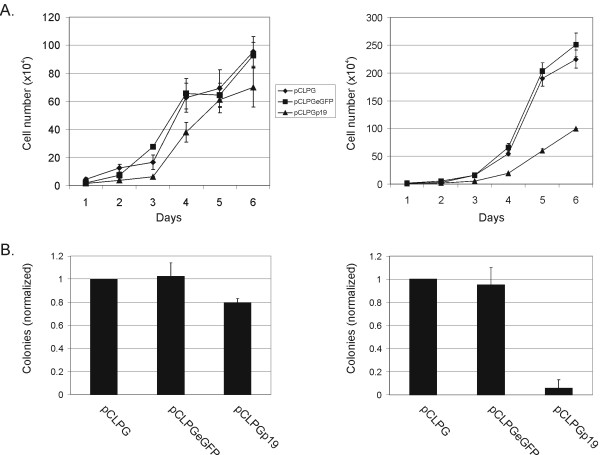
**B16 cell proliferation is resistant to 19Arf gene transfer**. (**A**) B16 (left) or C6 cells (right) were transduced with equal quantities of pCLPG (diamonds), pCLPGeGFP (squares) or pCLPGp19 (triangles) and replated for assessment of growth potential measured by counting cells each 24 hours during a 6 day period. Results presented are representative of three independent experiments. (**B**) B16 (left) or C6 cells (right) were transduced with equal quantities of pCLPG , pCLPGeGFP or pCLPGp19 and replated for the colony formation assay. The number of G418-resistant colonies counted in the pCLPG condition was considered as 1 and the colonies formed in the other conditions were normalized to this value. The results shown are the mean and standard deviation of three experiments.

In a growth curve assay, the p53-responsive pCLPG vectors were tested for their ability to inhibit the proliferation of B16 or C6 cells. Treatment of B16 cells with the pCLPGp19 vector did not confer a reduction in proliferation, but was successful for C6 (**Figure **5a). Alterations in growth were not detectable when either B16 or C6 cells were treated with pCLPGp53 or pCLp53, a retroviral vector with constitutive expression driven by the native LTR (data not shown).

Similarly, a colony formation assay showed no effect when B16 cells were transduced with pCLPGp19 as compared to the control, yet colony formation in C6 cells was efficiently reduced in the presence of pCLPGp19 (**Figure **5B). B16 cells were completely resistant to treatment with pCLPGp53 or pCLp53 in this assay (data not shown). In comparison, both pCLPGp53 and pCLp53, inhibited colony formation in C6 cells by about 50% (data not shown).

Since expression from the pCLPGp19 vector was reliable in both cell lines, yet each responded differently, we explored whether the barrier to proper p19Arf function involved the activation of p53.

### Combined pCLPGp19 and drug treatments induce p53 function in an additive manner

We set up a quantitative assay to measure the impact of combined gene transfer and drug treatment on p53 activity. For this, activity of p53 was measured in cells where pCLPGeGFP had been introduced to serve as a reporter and selected for G418 resistance. These cells were then transduced with a second pCLPG vector, subjected to drug treatments and eGFP reporter activity was quantified by flow cytometry.

**Figure 6:**
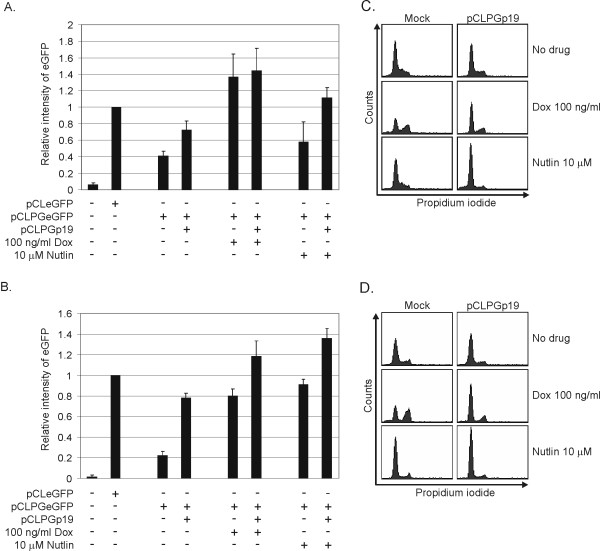
**Induction of endogenous p53 activity by combined gene and drug therapy**. Cells were transduced with pCLeGFP as a control or the p53-responsive retroviral reporter pCLPGeGFP and selected for G418 resistance. For the assays, the B16 (**A**) or C6 (**B**) reporter cells were either mock transduced or transduced with pCLPGp19, replated in 6-well dishes and then treated with drug as indicated. The following day, cells were harvested for flow cytometric analysis of eGFP expression. The mean eGFP intensity was determined by the FACS software and the value for pCLeGFP used for normalization. Shown is the average and standard deviation of duplicate samples performed in three independent experiments. Cell cycle analysis (**C **and **D**) was performed in parallel with the assays shown in A and B.

The introduction of pCLPGp19 resulted in weak induction of p53 activity in B16 cells, an approximate 1.75-fold increase as compared to the pCLPGeGFP reporter activity in the absence of either drug or genetic alteration (**Figure **6A). Pharmacologic induction of p53 activity could be achieved in B16 cells with doxorubicin, with or without pCLPGp19. In contrast, nutlin-3 treatment alone was not sufficient to stimulate significant p53 activity. Interestingly, p53-dependent reporter activity could be induced 2.5-fold by the combined treatment with pCLPGp19 and nutlin-3. This result shows that combining genetic and pharmacologic treatments had an additive effect in activating p53 in B16 cells.

In comparison, p53 activity in C6 cells was more efficiently induced by individual pCLPGp19 gene transfer or nutlin-3 treatments and yielded an additive effect when combined (**Figure **6B).

Cell cycle analysis was performed in parallel with the experiments described above (**Figure **6C and 6D). We observed that the combined treatment with pCLPGp19 plus drugs resulted in profoundly altered cell cycle patterns. For example, the combined treatment of B16 cells with pCLPGp19 and nutlin-3 resulted in a marked G1 arrest, whereas individual treatments produced little change in these cells. Treatment with doxorubicin produced a G2 arrest, yet the introduction of p19Arf in combination with doxorubicin yielded a G1 arrest. Cell cycle alterations in C6 cells followed a similar pattern, but with some subtle differences. In C6, treatment with nutlin-3 caused a pronounced G1 arrest that was slightly enhanced in the presence of pCLPG19.

We interpret these results as in indication that B16 cells are more resist to the activation of p53 than C6 when using either p19Arf gene transfer or nutlin-3 treatment. However, the combined treatments resulted in more highly activated p53 and marked cell cycle alterations.

### pCLPGp19 gene transfer pre-sensitizes cells to drug treatment

**Figure 7:**
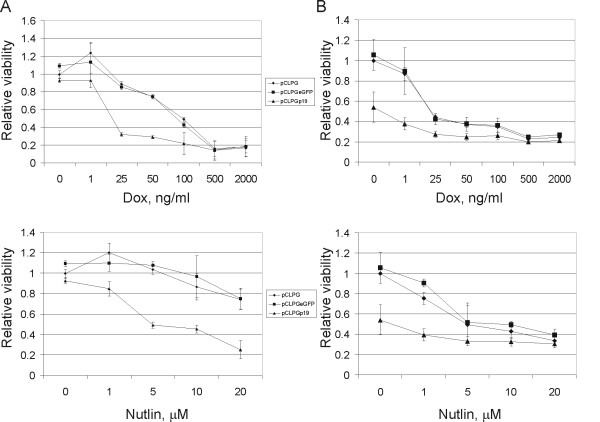
**pCLPGp19 gene transfer presensitizes cells to subsequent drug treatment as measured by an MTT cell viability assay**. B16 (**A**) or C6 cells (**B**) were transduced with equal quantities of pCLPG (diamonds), pCLPGeGFP (squares) or pCLPGp19 (triangles), replated in 96-well plates and quadruplicate samples were treated with drug as indicated for 48 hours before cell viability was assessed by MTT staining. The MTT absorbance observed in cells transduced with pCLPG, but without drug treatment, was used to normalize the values. Results represent the average and standard deviation of three independent experiments.

To determine whether the functional activation of p53 by the combination of pCLPGp19 gene transfer plus drug treatment is associated with a decrease in cell viability, we used a standard MTT assay. Cells were plated and then transduced with the pCLPG viruses. The next day, cells were collected, counted and replated in 96-well dishes. For these assays, drug treatments were allowed to proceed for 48 hours before MTT staining of viable cells. B16 cells previously transduced with pCLPGp19 were rendered more sensitive to treatment with either doxorubicin or nutlin-3 (**Figure **7A). Consistent with the previous assays, the combined treatment with p19Arf and nutlin-3 reduced the viability of B16 cells.

In C6 cells we observed that treatment with pCLPGp19 alone reduced viability by about 50%, consistent with the growth curve assays (**Figure **7B). Though C6 cells were quite sensitive to either nutlin-3 or doxorubicin, a subtle but consistent additional reduction in viability was seen by combining p19Arf gene transfer with pharmacologic therapies. So far, our results suggest that re-activating p53 by a combination of p19Arf gene transfer along with pharmacologic agents may present an interesting option for tumor cell inhibition, especially in cells that retain wild-type, but functionally inactive, p53.

### Combined p19Arf and nutlin-3 treatment induces death of B16 cells *in vivo*

**Figure 8:**
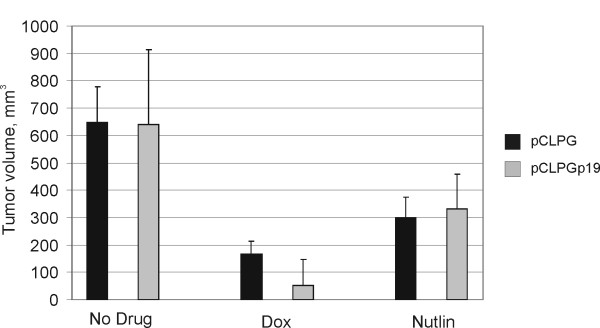
**Volume of B16 tumors**. B16 cells were transduced with either pCLPG (black bars) or pCLPGp19 (gray bars) and then treated with 25 ng/ml doxorubicin (Dox) or 10 μM nutlin-3, as indicated, before subcutaneous injection of 1 × 10^6 ^cells in C57BL/6 mice (n = 4/condition). After 15 days, the animals were sacrificed, tumors removed and measured. Tumor volume was determined using the formula V = π/6 × D1 × D2 × D3 (where D is the dimension in mm). Presented is the mean and standard deviation of the observed tumor volumes.

**Figure 9:**
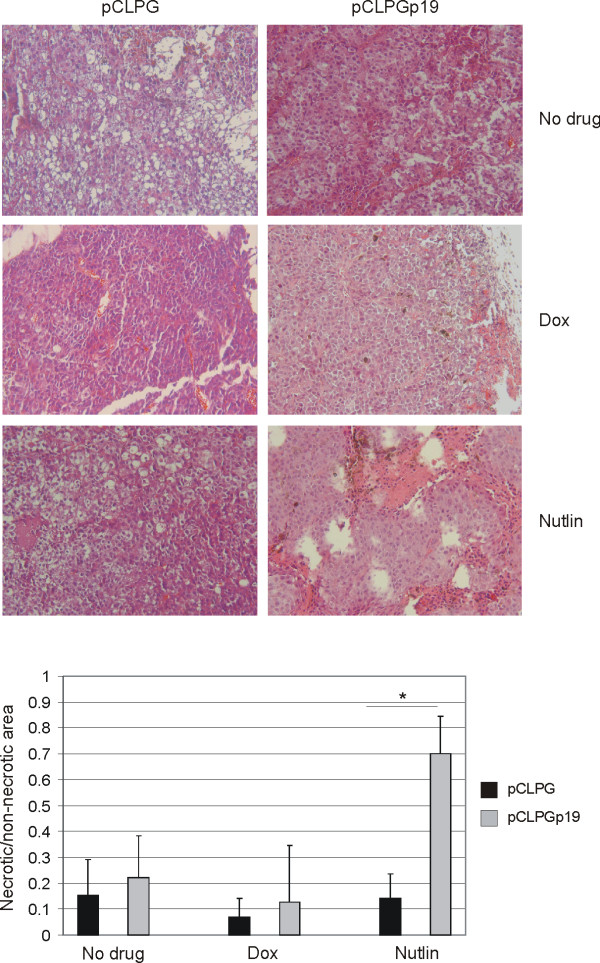
**Animal model of B16 tumor formation reveals increased necrosis when cells were treated with both pCLPGp19 and nutlin-3**. B16 cells were transduced *ex vivo *with pCLPG or pCLPGp19 followed by treatment with 25 ng/ml doxorubicin (dox) or 10 μM nutlin-3 (Nutlin) before subcutaneous implantation of 1 × 10^6 ^cells in C57BL/6 mice (n = 4/condition). After 15 days, the animals were sacrificed and tumors were analyzed histologically upon staining with HE. The ratio between necrotic and non-necrotic areas in photomicrographs of the HE sections was determined using ImageJ software and is presented as the mean and standard deviation of 15 to 20 fields from each of 3 sections from each tumor (*, student's t-test, p = 0.0012).

In an attempt to assess the impact of gene transfer and drug treatment in an animal model, B16 cells were transduced with pCLPG or pCLPGp19 and treated with doxorubicin or nutlin-3 *ex vivo*. Cells were then implanted subcutaneously in C57BL/6 mice and tumors were recovered on day 15. Reduction in tumor size was conferred by drug treatment, yet gene transfer did not to contribute to this result (**Figure **8). As shown in **Figure **9, tissue sections stained with HE revealed a statistically significant increase in areas of necrosis when the cells had been treated with the combination of pCLPGp19 gene transfer and nutlin-3, but not by either treatment alone (student's t-test, p = 0.0012).

**Figure 10:**
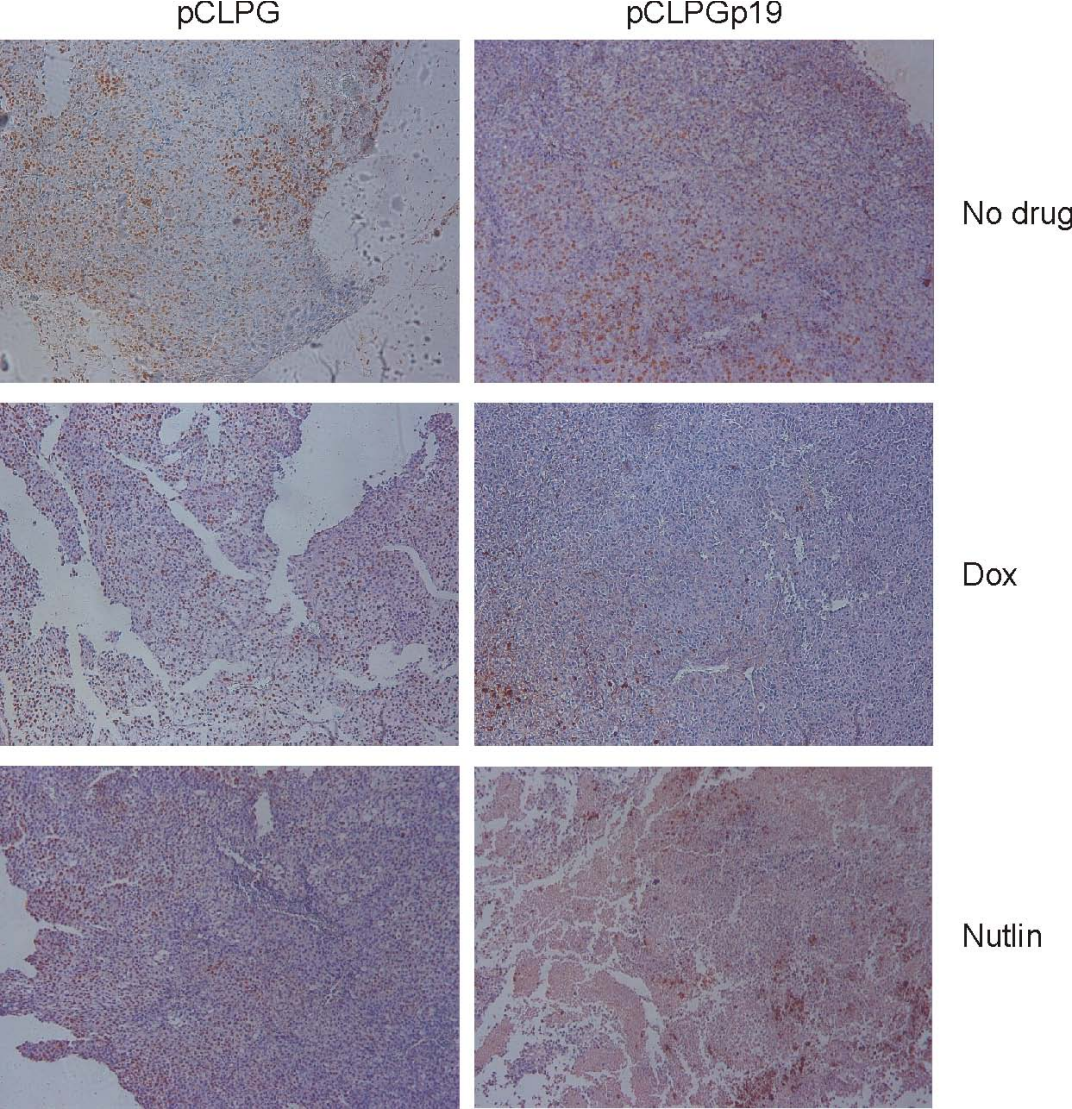
**Decreased proliferation, as revealed by BrdU incorporation, was associated with double treatment conditions**. Prior to sacrifice, animals were injected i.p. with a solution of BrdU. Paraffin embedded sections of tumors were probed with an anti-BrdU antibody conjugated to alkaline phosphatase and revealed through a peroxidase reaction.

Tumor cell proliferation was revealed by incorporation of BrdU prior to sacrifice followed by its immunohistochemical detection in the histologic sections. As shown in **Figure **10, BrdU staining was greatly reduced when the cells had been treated by combined pCLPGp19 gene transfer plus doxorubicin or nutlin-3 treatments, yet application of these treatments individually did not appreciably alter BrdU staining. TUNEL staining was performed, but no difference was observed among the experimental conditions (data not shown).

## Discussion

Both here and in previous studies [22,27], we saw that B16 and C6 are particularly resistant to p53 treatment. Since these cells harbor endogenous p53wt, they must possess a mechanism for maintaining p53 in an inactive state, such as the loss of p19Arf or over-expression of mdm2. Treatment approaches in this case include the re-activation of p53 by gene transfer or drug treatment. For example, introduction of p19Arf can activate p53 and complement its function. We set out to show that the p53-responsive pCLPG retroviral vector could be used to introduce p19Arf, uniting transgene function and control over its expression. That is to say, exogenous p19Arf should activate endogenous p53 and, in turn, reinforce expression from the pCLPG vector, resulting in even higher levels of exogenous p19Arf. We had hoped to observe that p19Arf gene transfer would be sufficient to inhibit proliferation, as was the case with C6 cells. In contrast, B16 cells were more resistant to the effects of p19Arf and required additional drug treatment in order to reduce viability.

Using a p53-responsive vector for the delivery of p19Arf to cancer cell lines that harbor p53wt established interplay between the vector, transgene and cellular components of the p53 pathway. However, the introduction of the pCLPGp19 vector resulted in distinct responses between the B16 and C6 cell lines. B16 cells were relatively resistant to either genetic or nutlin-3 treatments when applied individually, but their combination led to the activation of endogenous p53 and reduction in cell viability both in culture and in an animal model. In contrast, C6 cells were permissive to the activities of either p19Arf or nutlin-3 treatments as shown by the functional activation of endogenous p53.

The treatment of B16 cells with pCLPGp19 alone did not result in the reduction of proliferation, yet viral expression was reliable. Exogenous p19Arf was readily detected and was localized to the nucleolus. Since Northern and Western blots also confirmed viral expression, we proposed that p53 was not efficiently activated in B16 cells and that this may have been responsible for the continued proliferation of these cells. Holding p19Arf in the nucleolus due to its interaction with nucleophosmin is thought to prevent interaction of Arf with mdm2, which occurs in the nucleoplasm [28], though this is reversed when DNA damage is induced [29]. We observed that treatment with doxorubicin did not noticeably alter the localization of p19Arf (data not shown), though we used a relatively low drug dose. However, the treatment with the DNA-damaging agent doxorubicin was sufficient to activate p53 in both cell lines.

Gene transfer studies have shown that introduction of exogenous p14ARF could activate endogenous p53 wt more efficiently when both p14ARF and p53 were introduced simultaneously by adenoviral vectors [12,13]. Similarly, we observed that introduction of p19Arf was not sufficient to activate p53 in B16 cells. Interestingly, B16 cells were pre-sensitized to drug treatment by prior exposure to pCLPGp19. p19Arf gene transfer followed by treatment with nutlin-3 lead to markedly increased p53 activity, cell cycle alteration and reduced viability. Though C6 cells were more permissive to p19Arf or nutlin-3 treatments, their combination was beneficial in increasing the response to p53 stimulation. We propose that p53-responsive vectors may prove beneficial in functional and therapeutic studies of gene transfer in tumor models that present p53 wt, especially when combined with drug treatment.

Multiple pathways are involved in regulating p53 activity. Since C6 was sensitive to nutlin-3 treatment, we expect that p53 activity was squelched primarily through mdm2. In contrast, B16 cells probably possess additional mechanisms of abrogating p53 activity since these cells were quite resistant to nutlin-3. Reports in the literature point out that mdmx (mdm4) can render cells resistant to nutlin-3 treatment [17,18] and the interaction of p19Arf with mdmx is thought to inhibit mdmx activity [19,20]. Western blot analysis of mdmx in presence or absence of pCLPGp19, doxorubicin or nutlin-3 did not reveal any alteration in protein level or mobility (data not shown). Therefore, the mechanism for the resistance of B16 cells to nutlin-3 as well as the reason for p53 re-activation by the combination of p19Arf and nutlin-3 remains to be determined.

The treatment with either p19Arf or nutlin-3 should result in the neutralization of mdm2. However, each acts through a distinct mechanism. Nutlin-3 occupies the site on mdm2 where p53 would otherwise be bound. p19Arf, on the other hand, blocks the E3 ubiquitin ligase activity of mdm2. In this case, the influence of p19Arf may reach beyond p53 and include other factors with which mdm2 interacts, such as Hif1α [30] and PML [31].

Since treatment with pCLPGp19 was quite effective in C6 cells, we conclude that it is possible to use endogenous p53 to drive viral expression of p19Arf and bring about an increase in p53 activation and the concomitant reduction in cell proliferation. The resistance of B16 to p53 activation upon pCLPGp19 or nutlin-3 treatment may be particular for this cell line. However, B16 cells are widely studied as a model for melanoma and are particularly interesting since they can be studied in a syngeneic, immunocompetent animal model.

Analysis of tumor formation after *ex vivo *gene transfer and/or drug treatment revealed findings consistent with those described for the *in vitro *assays. Here too, inhibition of B16 proliferation and viability was revealed upon combined p19Arf gene transfer and nutlin-3 treatments. At the end of the 15 day observation period, tumor size was not a reliable indicator of the impact of treatment. However, a significant increase in necrotic tissue was revealed in tumors derived from B16 cells treated with both pCLPGp19 and nutlin-3. We did not find increased TUNEL staining upon gene transfer or drug treatments, possibly due to the kinetics of the treatment or due to the advanced stage of necrosis. A concomitant reduction in proliferation, as revealed by BrdU staining, was also seen in tumors arising from the doubly treated cells.

## Conclusions

In this study, we have explored the functional activation of endogenous p53 when p19Arf was introduced by a p53-responsive vector. In the absence of drug treatment, we observed reliable expression of exogenous p19Arf driven by endogenous p53. C6 cells were quite sensitive to either p19Arf gene transfer or nutlin-3 treatment alone and when combined these treatments yielded in additive effect. B16 cells were generally quite resistant to p19Arf or nutlin-3 treatments, though the combination of these resulted in markedly increased p53 activity as well as reduced viability. To the best of our knowledge, this work represents the first attempt to unite p19Arf gene transfer and nutlin-3 treatments. Here we have shown that combined treatments activated p53 and reduced the viability of B16 cells.

## Competing interests

The authors declare that they have no competing interests.

## Authors' contributions

CAM carried out the animal studies and histologic analyses; RBSS performed the northern and western blot analysis; ACPVC performed the immunofluorescence study and provided technical assistance; DBZ, MCB and PF performed FACS analysis and provided technical assistance; ECS participated in study design and helped draft the manuscript; BES conceived of the study, participated in its design and coordination and drafted the manuscript. All authors read and approved the final manuscript.

## Pre-publication history

The pre-publication history for this paper can be accessed here:

http://www.biomedcentral.com/1471-2407/10/316/prepub

## References

[B1] Kastan MB Wild-type p53: tumors can't stand it Cell 2007 128 837 840 10.1016/j.cell.2007.02.022 17350571

[B2] Martins CP Brown-Swigart L Evan GI Modeling the therapeutic efficacy of p53 restoration in tumors Cell 2006 127 1323 1334 10.1016/j.cell.2006.12.007 17182091

[B3] Ventura A Kirsch DG McLaughlin ME Tuveson DA Grimm J Lintault L Newman J Reczek EE Weissleder R Jacks T Restoration of p53 function leads to tumour regression in vivo Nature 2007 445 661 665 10.1038/nature05541 17251932

[B4] Xue W Zender L Miething C Dickins RA Hernando E Krizhanovsky V Cordon-Cardo C Lowe SW Senescence and tumour clearance is triggered by p53 restoration in murine liver carcinomas Nature 2007 445 656 660 10.1038/nature05529 17251933 PMC4601097

[B5] Giglia-Mari G Sarasin A TP53 mutations in human skin cancers Hum Mutat 2003 21 217 228 10.1002/humu.10179 12619107

[B6] Polsky D Bastian BC Hazan C Melzer K Pack J Houghton A Busam K Cordon-Cardo C Osman I HDM2 protein overexpression, but not gene amplification, is related to tumorigenesis of cutaneous melanoma Cancer Res 2001 61 7642 7646 11606406

[B7] Sharpless E Chin L The INK4a/ARF locus and melanoma Oncogene 2003 22 3092 3098 10.1038/sj.onc.1206461 12789286

[B8] Ohgaki H Kleihues P Genetic pathways to primary and secondary glioblastoma Am J Pathol 2007 170 1445 1453 10.2353/ajpath.2007.070011 17456751 PMC1854940

[B9] Fulci G Labuhn M Maier D Lachat Y Hausmann O Hegi ME Janzer RC Merlo A Van Meir EG p53 gene mutation and ink4a-arf deletion appear to be two mutually exclusive events in human glioblastoma Oncogene 2000 19 3816 3822 10.1038/sj.onc.1203700 10949938

[B10] Kleihues P Ohgaki H Primary and secondary glioblastomas: from concept to clinical diagnosis Neuro Oncol 1999 1 44 51 10.1215/15228517-1-1-44 11550301 PMC1919466

[B11] Shangary S Wang S Targeting the MDM2-p53 interaction for cancer therapy Clin Cancer Res 2008 14 5318 5324 10.1158/1078-0432.CCR-07-5136 18765522 PMC2676446

[B12] Lu W Lin J Chen J Expression of p14ARF overcomes tumor resistance to p53 Cancer Res 2002 62 1305 1310 11888896

[B13] Tango Y Fujiwara T Itoshima T Takata Y Katsuda K Uno F Ohtani S Tani T Roth JA Tanaka N Adenovirus-mediated p14ARF gene transfer cooperates with Ad5CMV-p53 to induce apoptosis in human cancer cells Hum Gene Ther 2002 13 1373 1382 10.1089/104303402760128595 12162819

[B14] Huang Y Tyler T Saadatmandi N Lee C Borgstrom P Gjerset RA Enhanced tumor suppression by a p14ARF/p53 bicistronic adenovirus through increased p53 protein translation and stability.[erratum appears in Cancer Res. 2003 Aug 15;63(16):5171] Cancer Research 2003 63 3646 3653 12839954

[B15] Vassilev LT Vu BT Graves B Carvajal D Podlaski F Filipovic Z Kong N Kammlott U Lukacs C Klein C Fotouhi N Liu EA In vivo activation of the p53 pathway by small-molecule antagonists of MDM2 Science 2004 303 844 848 10.1126/science.1092472 14704432

[B16] Vassilev LT p53 Activation by small molecules: application in oncology J Med Chem 2005 48 4491 4499 10.1021/jm058174k 15999986

[B17] Wade M Wong ET Tang M Stommel JM Wahl GM Hdmx modulates the outcome of p53 activation in human tumor cells J Biol Chem 2006 281 33036 33044 10.1074/jbc.M605405200 16905769

[B18] Hu B Gilkes DM Farooqi B Sebti SM Chen J MDMX overexpression prevents p53 activation by the MDM2 inhibitor Nutlin J Biol Chem 2006 281 33030 33035 10.1074/jbc.C600147200 16905541

[B19] Ghosh M Weghorst K Berberich SJ MdmX inhibits ARF mediated Mdm2 sumoylation Cell Cycle 2005 4 604 608 15876864

[B20] Jackson MW Lindstrom MS Berberich SJ MdmX binding to ARF affects Mdm2 protein stability and p53 transactivation J Biol Chem 2001 276 25336 25341 10.1074/jbc.M010685200 11297540

[B21] Strauss BE Costanzi-Strauss E pCLPG: a p53-driven retroviral system Virology 2004 321 165 172 10.1016/j.virol.2003.12.021 15051377

[B22] Strauss BE Bajgelman MC Costanzi-Strauss E A novel gene transfer strategy that combines promoter and transgene activities for improved tumor cell inhibition Cancer Gene Ther 2005 12 935 946 10.1038/sj.cgt.7700846 15905860

[B23] DuBridge RB Tang P Hsia HC Leong PM Miller JH Calos MP Analysis of mutation in human cells by using an Epstein-Barr virus shuttle system Mol Cell Biol 1987 7 379 387 3031469 10.1128/mcb.7.1.379-387.1987 PMC365079

[B24] Naviaux RK Costanzi E Haas M Verma IM The pCL vector system: rapid production of helper-free, high-titer, recombinant retroviruses Journal of Virology 1996 70 5701 5705 8764092 10.1128/jvi.70.8.5701-5705.1996 PMC190538

[B25] Bajgelman MC Costanzi-Strauss E Strauss BE Exploration of critical parameters for transient retrovirus production J Biotechnol 2003 103 97 106 10.1016/S0168-1656(03)00103-2 12814868

[B26] Bajgelman MC Strauss BE The DU145 human prostate carcinoma cell line harbors a temperature-sensitive allele of p53 Prostate 2006 66 1455 1462 10.1002/pros.20462 16741917

[B27] Strauss BE Fontes RB Lotfi CF Skorupa A Bartol I Cipolla-Neto J Costanzi-Strauss E Retroviral transfer of the p16INK4a cDNA inhibits C6 glioma formation in Wistar rats Cancer Cell Int 2002 2 2 10.1186/1475-2867-2-2 11983028 PMC116432

[B28] Gjerset RA DNA damage, p14ARF, nucleophosmin (NPM/B23), and cancer J Mol Histol 2006 37 239 251 10.1007/s10735-006-9040-y 16855788

[B29] Lee C Smith BA Bandyopadhyay K Gjerset RA DNA damage disrupts the p14ARF-B23(nucleophosmin) interaction and triggers a transient subnuclear redistribution of p14ARF Cancer Res 2005 65 9834 9842 10.1158/0008-5472.CAN-05-1759 16267006

[B30] Ravi R Mookerjee B Bhujwalla ZM Sutter CH Artemov D Zeng Q Dillehay LE Madan A Semenza GL Bedi A Regulation of tumor angiogenesis by p53-induced degradation of hypoxia-inducible factor 1alpha Genes Dev 2000 14 34 44 10640274 PMC316350

[B31] Wei X Yu ZK Ramalingam A Grossman SR Yu JH Bloch DB Maki CG Physical and functional interactions between PML and MDM2 J Biol Chem 2003 278 29288 29297 10.1074/jbc.M212215200 12759344

